# Myocardial extravascular extracellular volume fraction measurement by gadolinium cardiovascular magnetic resonance in humans: slow infusion versus bolus

**DOI:** 10.1186/1532-429X-13-16

**Published:** 2011-03-04

**Authors:** Erik B Schelbert, Stephen M Testa, Christopher G Meier, William J Ceyrolles, Joshua E Levenson, Alexander J Blair, Peter Kellman, Bobby L Jones, Daniel R Ludwig, David Schwartzman, Sanjeev G Shroff, Timothy C Wong

**Affiliations:** 1 Department of Medicine, University of Pittsburgh School of Medicine, Pittsburgh, PA, USA; 2 UPMC Cardiovascular Magnetic Resonance Center, Pittsburgh, PA, USA; 3 Cardiovascular Institute, UPMC, Pittsburgh, PA, USA; 4 Carnegie Mellon University, Pittsburgh, PA, USA; 5 National Heart, Lung, Blood Institute, Bethesda, MD, USA; 6 Center for Research on Health Care, University of Pittsburgh School of Medicine, Pittsburgh, PA, USA; 7 Department of Bioengineering, University of Pittsburgh, Pittsburgh, PA, USA

## Abstract

**Background:**

Myocardial extravascular extracellular volume fraction (Ve) measures quantify diffuse fibrosis not readily detectable by conventional late gadolinium (Gd) enhancement (LGE). Ve measurement requires steady state equilibrium between plasma and interstitial Gd contrast. While a constant infusion produces steady state, it is unclear whether a simple bolus can do the same. Given the relatively slow clearance of Gd, we hypothesized that a bolus technique accurately measures Ve, thus facilitating integration of myocardial fibrosis quantification into cardiovascular magnetic resonance (CMR) workflow routines. Assuming equivalence between techniques, we further hypothesized that Ve measures would be reproducible across scans.

**Methods:**

In 10 volunteers (ages 20-81, median 33 yr, 3 females), we compared serial Ve measures from a single short axis slice from two scans: first, during a constant infusion, and second, 12-50 min after a bolus (0.2 mmol/kg gadoteridol) on another day. Steady state during infusion was defined when serial blood and myocardial T1 data varied <5%. We measured T1 on a 1.5 T Siemens scanner using a single-shot modified Look Locker inversion recovery sequence (MOLLI) with balanced SSFP. To shorten breath hold times, T1 values were measured with a shorter sampling scheme that was validated with spin echo relaxometry (TR = 15 sec) in CuSO4-Agar phantoms. Serial infusion vs. bolus Ve measures (n = 205) from the 10 subjects were compared with generalized estimating equations (GEE) with exchangeable correlation matrices. LGE images were also acquired 12-30 minutes after the bolus.

**Results:**

No subject exhibited LGE near the short axis slices where Ve was measured. The Ve range was 19.3-29.2% and 18.4-29.1% by constant infusion and bolus, respectively. In GEE models, serial Ve measures by constant infusion and bolus did not differ significantly (difference = 0.1%, p = 0.38). For both techniques, Ve was strongly related to age (p < 0.01 for both) in GEE models, even after adjusting for heart rate. Both techniques identically sorted older individuals with higher mean Ve values.

**Conclusion:**

Myocardial Ve can be measured reliably and accurately 12-50 minutes after a simple bolus. Ve measures are also reproducible across CMR scans. Ve estimation can be integrated into CMR workflow easily, which may simplify research applications involving the quantification of myocardial fibrosis.

## Introduction

Emerging techniques in cardiovascular magnetic resonance (CMR) offer new and important opportunities to quantify pathologic myocardial fibrosis but require further understanding to optimize their integration into CMR workflow routines. Myocardial fibrosis, characterized by expansion of the myocardial extracellular matrix and accumulation of interstitial collagen, is the hallmark of pathologic remodeling [1]. This derangement alters electrical and mechanical function. Extracellular collagen, the principal constituent of the expanded extracellular matrix [1], blocks electrical conduction [2-7], constitutes the substrate for reentry and lethal ventricular arrhythmia [8], and stiffens the myocardium [1].

While CMR with late gadolinium enhancement (LGE) can detect large macroscopic foci of myocardial fibrosis [9], it cannot detect the full spectrum of myocardial fibrosis. LGE relies on relative differences in signal intensities expressed in arbitrary units and employs the myocardium with lowest signal intensity as a reference for normality, regardless of the degree of fibrosis contained within. Two lines of evidence underscore a need for alternative techniques beyond LGE. First, a recent study indicated that collagen biomarkers were more sensitive than LGE for detecting early myocardial fibrosis in hypertrophic cardiomyopathy [10]. Second, roughly half of the excess collagen in the setting of dilated cardiomyopathy is deposited diffusely between myocytes as opposed to focally [11], therefore undetectable by LGE.

Standard extracellular gadolinium (Gd) contrast tracks collagen at the microscopic level with high fidelity without an apparent critical mass needed for detection [12], thereby offering an opportunity to use Gd concentration as a robust indicator of myocardial fibrosis burden. Indeed, recently developed methods [13-15] assess the volume fraction of extravascular extracellular matrix (Ve) in human myocardium (with standard contrast agents) by exploiting the linear relationship between change in relaxivity and Gd concentration. These methods, however, require steady state equilibrium between the plasma and myocardial interstitium for successful Ve measurement. Whether a lengthy continuous intravenous infusion is needed to achieve steady state, or whether a simple bolus is sufficient to achieve steady state equilibrium remains uncertain, but an important issue for several reasons: First, introducing a lengthy infusion imposes additional burden on patients and considerable constraints on routine CMR workflow routines. Second, these data are important to interpret results from studies employing either the bolus [13,15] or infusion [14] techniques. Third, if the Gd dose reserved for LGE is lowered (e.g., halving a 0.2 mmol/kg dose to 0.1 mmol/kg bolus)[14] to allocate a portion for the subsequent infusion, the sensitivity of LGE may decline since doses as low as 0.1 mmol/kg have reduced sensitivity for myocardial infarction [16].

Given the slow renal clearance of Gd, its low molecular weight, and rapid dispersion throughout tissues [17,18], we tested whether a simple bolus permits accurate Ve measurement. We hypothesized that the equilibrium between plasma and the interstitium contrast concentrations would be maintained during slow renal clearance so that the ratios of Gd in myocardium and blood, the change in their respective relaxivities, and Ve would remain constant and similar to measures acquired during steady state infusion. If indeed Ve measurement was not related to technique, we further hypothesized that Ve measures would also be reproducible between scans. We compared the accuracy of serial Ve measures from two different CMR scans (at 1.5 T) in each subject, the first involving a continuous infusion to demonstrate the attainment of steady state equilibrium, and the second involving a simple intravenous bolus as is typically performed for LGE imaging.

## Methods

### T1 and T2 Measurement in Phantoms

CMR-based Ve measurement requires robust measurement of T1. Therefore, we compared 2 different methods of T1 measurement in phantoms with varying concentrations of CuSO4 and Agar to produce similar T1 and T2 of myocardium and blood, before and after Gd administration. Physiologic T2 values are important for sequences with steady state free precession readouts because signal intensity varies by T2/T1. All measurements were acquired with a 1.5 Tesla Siemens Magnetom Espree (Siemens Medical Solutions, Erlangen, Germany) with a 32 channel phased array cardiovascular coil. To obtain T1 values from CMR data, we used a 3 parameter model to describe signal intensity (SI) as a function of inversion time (TI): SI = | A - B·e^(-TI/T1*) ^|, where T1 = T1*((B/A)-1) [19]. T2 values were obtained using a 2 parameter model describing SI as a function of echo time (TE): SI = A·e^(-TE/T2)^. Least square estimates of model parameters were obtained using the Levenberg-Marquardt algorithm in Matlab^® ^(The MathWorks, Inc., Natick, Massachusetts).

#### T1 and T2 Relaxometry

For a reference standard, T1 was measured by spin echo inversion recovery experiments, acquiring 1 k-space line per readout (i.e., echo train = 1), TR = 15 s, TE = 10 msec, FA 90 degrees, with 10 inversion times ranging from 25 - 8000 msec. T2 was measured by spin echo experiments, acquiring 1 k-space line per readout, TR = 15 s, FA 90 degrees, with 10 TE times ranging from 10 - 400 msec.

#### T1 using MOLLI

We measured T1 using an ECG-gated single-shot modified Look Locker inversion recovery (MOLLI) sequence as described by Messroghli et al. [20] with simulated heart rates. Typical parameters were FOV 360 × 270 cm, matrix 256 × 128, 6 mm thickness, FA 35 degrees, 6/8 partial Fourier k-space sampling, parallel processing factor (GRAPPA) 2, and initial effective inversion time (TIeff) 90 msec with a TIeff increment of 80 msec. To sample T1 recovery, serial single shot diastolic images were acquired every heart beat after 3 nonselective adiabatic inversion pulses (i.e., 3, 3, and 5 images after each inversion pulse, totaling 11 images), and 3 dummy heart beats separated inversion pulses [20]. We refer to this sampling approach as "Classic MOLLI."

We also used a shorter sampling scheme to minimize breath hold times. A shorter sampling scheme may also minimize potential T1 measurement error due to effects related to repeated excitations prior to complete recovery of magnetization between serial inversion pulses. For longer *precontrast T1 recovery *of myocardium and blood (~950 and 1500 msec, respectively), we adjusted the above sampling scheme to employ only 2 nonselective adiabatic inversion pulses with a 5 and 1 sampling scheme (6 images total).

For *post contrast T1 recovery *that demonstrates faster relaxation rates, we employed 3 inversion pulses with a 4, 2, and 1 sampling scheme (7 images total). This approach attempted to ensure adequate sampling of the initial portion of the T1 recovery curve where the rate of relaxation was highest given the constraints imposed by sampling limited to diastole. Hence, additional data points were sampled during early T1 recovery for post contrast T1 measures. Furthermore, in contrast to precontrast sampling, we reasoned that the sample from 5^th ^heart beat was unnecessary because most post contrast T1 values were approximately 500 msec or less, meaning that 99% of the magnetization had recovered by four heart beats (i.e., 2.5 sec, or 5 multiples of post contrast T1 if the R-R interval was ≥625 msec). We refer to this overall shortened T1 sampling approach adapted to the anticipated precontrast or post contrast T1 recovery curve as "Hybrid MOLLI." Using "Classic" and "Hybrid" MOLLI, we compared T1 data from phantoms against the spin echo derived values at slower (68 bpm) and faster simulated heart rates (92 bpm). Each of the 7 phantoms had 5 measurements by each MOLLI technique.

### Monte Carlo Simulation

To explore how variable sampling of the T1 recovery curve affected the precision of T1 measures, we used Monte Carlo simulations to compare the root mean square error associated with the "Classic" and "Hybrid" sampling schemes mentioned above. Importantly, this simulation did not measure effects related to incomplete magnetization recovery between serial excitation pulses (whereas the phantom studies did), and thus was not used to assess accuracy. For simulation, we assumed a maximum signal amplitude (A.U.) of 250 and added noise with a standard deviation of 10. We performed 128 trials for each set of parameters, varying the heart rate from 50-100 beats per minute (5 bpm increments) and sampled T1 ranges from 200-500 msec and 800-1500 msec (100 msec increments).

### Subjects

After Institutional Review Board approval and written informed consent, ten volunteers received CMR exams. Six were young adults who were under age 35, healthy, without symptoms or known comorbidity. Since renal function declines with age [21], these young subjects represented those with the highest renal function, fastest Gd clearance, and thus the greatest potential to disrupt equilibrium (and also Ve measures) after a bolus. Data were also acquired from four older subjects with ages ranging from 66-81 years, all clinically stable and asymptomatic, but with significant comorbidity. These older subjects represented those with a potential to disrupt equilibrium not from robust renal clearance, but from 1) a larger volume of distribution for Gd contrast, and 2) less capillary density [22-24] which occurs with older age and associated comorbidity. Together, the larger interstitial reservoir for Gd to accumulate and the diminished capillary density might prevent steady state equilibrium between plasma and myocardium after a bolus.

### T1, lambda, and Ve measurement in Subjects

#### Infusion

We modified the intravenous infusion protocol outlined by Thornhill et al. [25] to constrain the total contrast dose to be 0.2 mmol/kg. For the infusion CMR scan, we gave a loading dose (0.1 mmol/kg gadoteridol bolus (Prohance, Bracco Diagnostics, Princeton, NJ)) followed by a 200 mL/hr infusion for ~1 hr with 0.1 mmol/kg diluted in 200 mL saline. Assuming a baseline myocardial blood flow of ~1 mL/g/min and an extraction efficiency of 0.6, this strategy should attain plasma-interstitium equilibrium for a wide range of Ve by ~25 minutes [26]. We arrived at the final infusion protocol by further iterative adjustments in 4 additional volunteers. T1 measures were obtained precontrast and repetitively during the 1-hour period after the initiation of contrast infusion. We defined steady state equilibrium during infusion when T1 values of both blood and myocardium varied by <5% [14].

#### Bolus

For the bolus CMR scan acquired on a different day, subjects received a 0.2 mmol/kg intravenous bolus of gadoteridol. T1 measures were obtained precontrast and repetitively during the 12-50 min period after the receipt of the contrast bolus. We delayed Ve measurement for 12 minutes after the bolus to ensure full dispersion and equilibration of Gd contrast between tissues, which occurs after at least 5 minutes [13].

#### Ve measurement

We measured Ve as outlined by Jerosch-Herold et al. [13] Specifically,

Ve=[λ⋅ρ⋅(1-hematocrit)]-Vp

where Ve is the myocardial extravascular extracellular volume fraction, ρ is the specific density of myocardial tissue (1.05), Vp is the myocardial plasma volume fraction (assumed to be a constant 0.045 [13,27-29], reflecting capillary density), and λ=[ΔR1_myocardium_]/[ΔR1_bloodpool_] pre and post Gd contrast (where R1 = 1/T1) [30,31]. Hematocrit was measured from blood samples drawn at the time of each CMR session. We carefully localized one mid ventricular short axis slice per subject for T1 measurement to match slice position across different scans. Blood pool regions of interest were intentionally large to average any inhomogeneity related to diastolic blood flow. Images were analyzed with Siemens multimodality workstations. When drawing circumferential regions of interest in the myocardium with computer assisted planimetry, care was taken to avoid the very edges of myocardium to avoid contaminating the measurement with partial volume averaging from voxels straddling the myocardial-blood pool border [12].

### Late Gadolinium Enhancement

We acquired late gadolinium enhancement images 10-30 minutes after the 0.2 mmol/kg gadoteridol bolus in all subjects in the usual fashion [32]. To optimize detection of grossly evident scar or myocardial infarction, we used a phase sensitive segmented gradient echo inversion recovery [33] pulse sequence to increase signal to noise ratios, correct for surface coil intensity variation, and render signal intensity proportional to T1 recovery.

### Statistical Analysis

T-tests compared continuous variables. Linear regression compared the mean Ve for subjects (given unequal number of measurements for bolus and infusion) according to measurement technique; Bland-Altman plots assessed overall agreement and bias between Ve measurement techniques [34]. Generalized estimating equations (GEE) with exchangeable correlation matrices to account for serial measures compared the linear regression slopes of individual classic and hybrid MOLLI T1 measurements against spin echo T1 measurements. GEE also summarized any differences between the infusion (at steady state equilibrium) and bolus techniques for Ve measurement, adjusting for repeated Ve measures across individuals over time. To assess for an interaction between time and Ve measures, a time variable and a variable representing the product of Ve and time was entered into the model. Analyses were performed using SAS (Cary, NC) and Microsoft Excel (Redmond, Washington).

## Results

### Monte Carlo Simulation

Monte Carlo simulation revealed no difference in the root mean square error (RMSE) between classic and hybrid MOLLI for T1 values between 200-500 msec (RMSE 14 vs. 14, p = 0.51, paired t test). At higher T1 values of 800-1500 msec, classic MOLLI exhibited better precision than hybrid MOLLI (RMSE 29 vs. 45, respectively, p < 0.001). It should be noted that this simulation scheme did not account for effects related to repeated excitation pulses as did the phantom data below.

### Phantom Studies

Phantom T1 and T2 values measured by spin echo relaxometry closely approximated the expected range of physiologic values before and after contrast administration. The phantom representing blood before contrast had T1/T2 values of 1535 msec/172 msec. Two precontrast myocardium phantoms had T1/T2 values of 909 msec/44 msec and 887 msec/38 msec. Postcontrast blood phantoms had T1/T2 values of 309 msec/187 msec and 251 msec/171 msec. Postcontrast myocardial phantoms had T1/T2 values of 342 msec/53 msec and 260 msec/53 msec.

**Figure 1:**
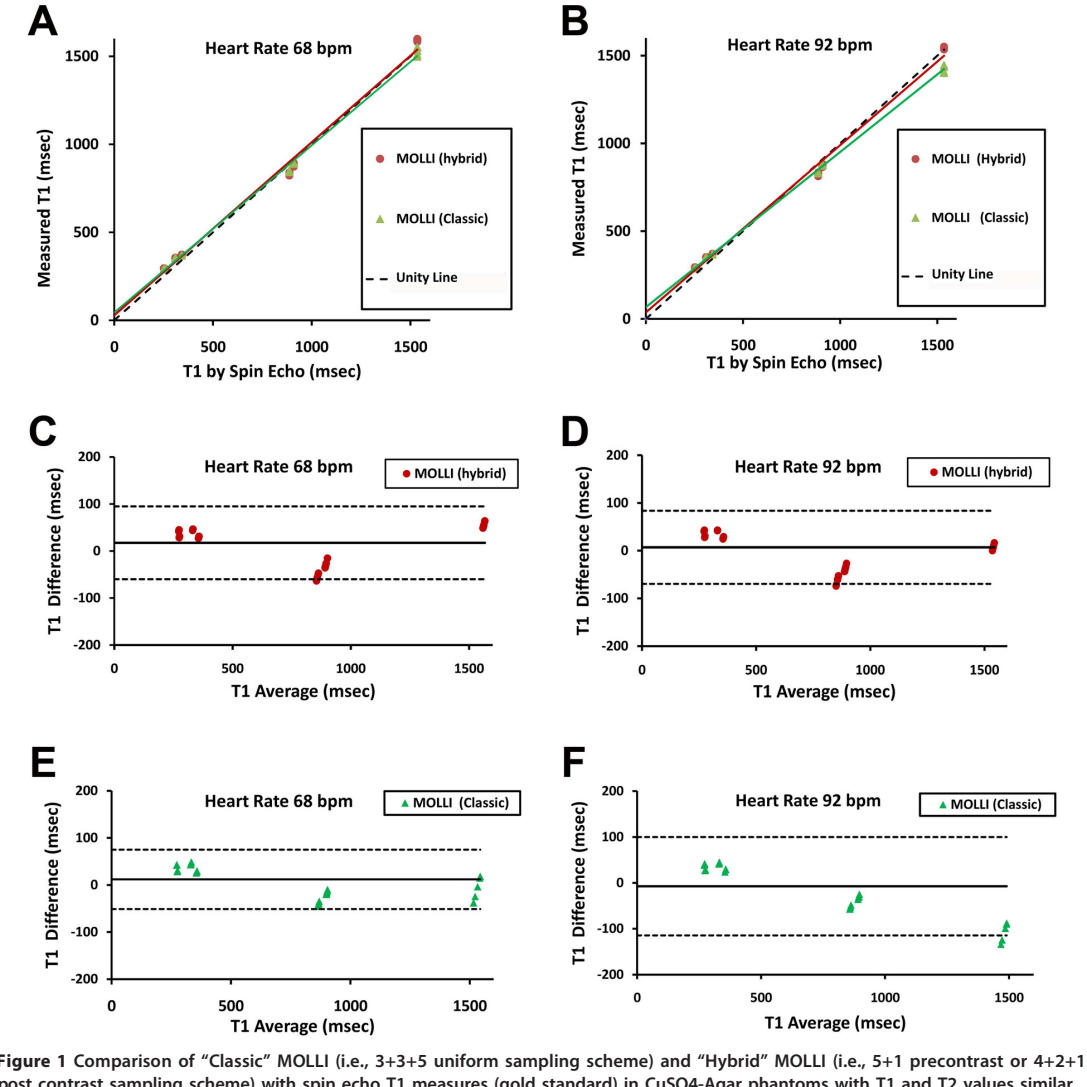
**Comparison of "Classic" MOLLI (i.e., 3+3+5 uniform sampling scheme) and "Hybrid" MOLLI (i.e., 5+1 precontrast or 4+2+1 post contrast sampling scheme) with spin echo T1 measures (gold standard) in CuSO4-Agar phantoms with T1 and T2 values similar to myocardium and blood before and after contrast (see text for details)**. Both exhibit excellent correlation with spin echo measures. "Hybrid MOLLI" exhibits slightly better correlation (panels A and B) and better agreement with less bias (panels C and D) compared to "classic" MOLLI (Panels E and F), especially at higher heart rates. Each of the 7 phantoms had 5 serial measurements.

Correlation plots and Bland-Altman analysis demonstrated that hybrid MOLLI exhibited better agreement with spin echo T1 relaxometry measures than classic MOLLI. At a relatively low heart rate of 68 bpm, serial T1 measures (5 repetitions per phantom for each technique; thus 70 total measures) by ECG gated MOLLI were highly correlated with the spin echo 'gold standard' measures (Figure 1). The regression line for hybrid MOLLI had a slope of 0.98 (95%CI 0.91-1.06), that was not significantly different from unity (p = 0.66). In contrast, the regression line for classic MOLLI had a slope of 0.95 (95%CI 0.91-0.99), significantly less than unity (p = 0.007). These differences were more pronounced at higher heart rates (i.e., 92 bpm) where the regression line slopes for hybrid and classic MOLLI were 0.95 (95% CI 0.89-1.01; p = 0.12) and 0.88 (95% CI 0.87-0.89; p < 0.001), respectively. At higher heart rates, Bland-Altman analysis revealed that the classic MOLLI approach underestimated T1 when T1 was long but overestimated T1 when T1 was short. This bias compresses the dynamic range of the T1 differences. Hybrid MOLLI exhibited less bias and generally yielded better agreement.

**Figure 2:**
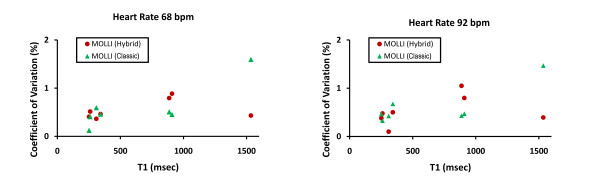
**Coefficients of variation for phantoms measured by "Classic" MOLLI and "Hybrid" MOLLI techniques (see text for details)**. Both techniques exhibit excellent reproducibility (<2%), but the hybrid MOLLI technique exhibits more precision at higher T1 values.

**Figure 3:**
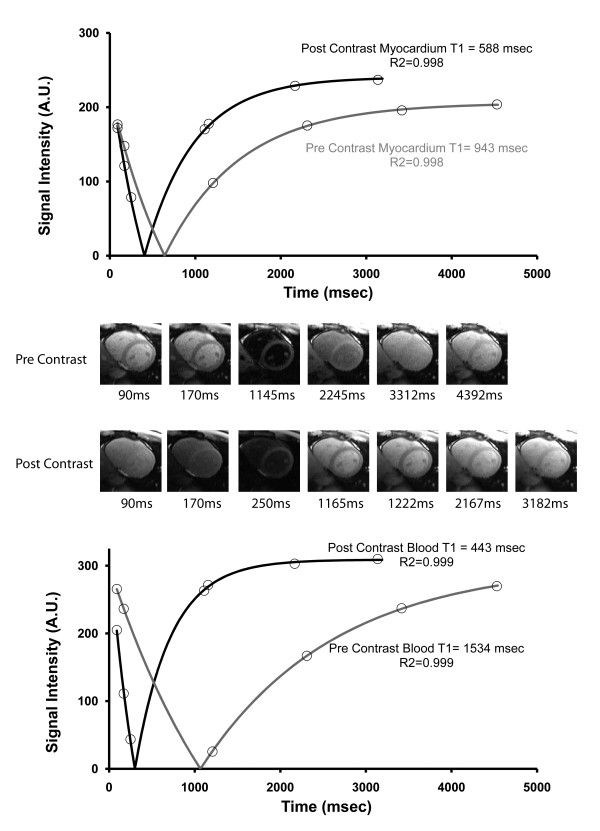
**Representative T1 values of myocardium and blood in a subject before and after contrast can be measured with a modified Look Locker pulse sequence employing single shot SSFP imaging within a single breath hold**. Signal intensities are plotted against effective inversion time. A three parameter fitting of the data with a correction for read-out attenuation yields T1 values (see text for details). The top panel shows myocardial T1 curves before and after contrast whereas the lower panel shows the blood T1 curves before and after contrast. Most of the magnetization recovers after 4 heart beats *after *contrast administration. Pre and post contrast thumbnails corresponding to the myocardium and blood pool data points in the curves are also shown in order of effective inversion times.

Both the hybrid and classic MOLLI acquisitions demonstrated excellent reproducibility for repeated measurements. The coefficients of variation for all phantoms were <1.6%. At a heart rate of 68 bpm the mean coefficient of variation was 0.55% and 0.59% for hybrid versus classic MOLLI, respectively; at a heart rate of 92 bpm the mean coefficient of variation was 0.53% and 0.61% for hybrid versus classic MOLLI, respectively. The coefficients of variation for each phantom measured by either technique are plotted as a function of T1 for both a slow and faster heart rate in Figure 2. Given the better correlation and agreement with T1 measurement compared to the spin echo gold standard as well as the comparable precision in phantom studies (but not simulation studies), hybrid MOLLI techniques were selected for T1 measurement in human subjects as demonstrated in Figure 3.

### Subjects

Median age of the 10 subjects was 33 years (range 20-81 yrs), and 3 were female. All subjects were free of cardiac symptoms. Characteristics of the subjects are shown in Table 1. The six younger subjects were healthy without any associated comorbidity. No subject exhibited abnormalities on late gadolinium enhancement images in the slice position used for Ve measurement; one subject with a history of myocardial infarction exhibited late gadolinium enhancement remote from myocardium used for Ve measurement.

**Table 1:** Characteristics of subjects undergoing assessment of the extravascular extracellular volume fraction (Ve)

Age	Gender	Comorbidity	Mean Infusion λ (SEM)	Infusion hematocrit (%)	Mean Bolus λ (SEM)	Bolus hematocrit (%)
20	male	none	0.452 (0.002)	47.7	0.424 (0.007)	46.1
21	male	none	0.399 (0.002)	41.2	0.392 (0.004)	41.9
21	female	none	0.434 (0.004)	39.6	0.424 (0.004)	38.9
22	male	none	0.398 (0.002)	41.0	0.401 (0.003)	42.1
31	male	none	0.412 (0.002)	40.4	0.424 (0.003)	42.3
34	male	none	0.437 (0.001)	42.7	0.448 (0.002)	41.5
66	female	Ulcerative colitis, colectomy, diabetes, left bundle branch block, recurrent infections	0.451 (0.002)	30.0	0.442 (0.002)	29.8
68	female	Diabetes, obesity, hypertension	0.453 (0.002)	38.0	0.461 (0.003)	38.9
69	male	Myocardial infarction, hypertension	0.473 (0.006)	44.4	0.474 (0.009)	44.0
81	male	Atrial fibrillation	0.460 (0.002)	41.2	0.459 (0.004)	40.4

Subjects were either healthy young adults or older individuals with significant comorbidity.

Representative images used for T1 measurement are shown in Figure 3. Signal intensity versus inversion time plots indicate good fit to the T1 recovery curves. Post contrast T1 curves for blood and myocardium indicate magnetization has nearly completely recovered after 4 heart beats (hence the 4+2+1 "hybrid" post contrast sampling scheme). Before contrast, 5 heart beats are needed to sample the full span of the longer T1 recovery curve.

**Figure 4:**
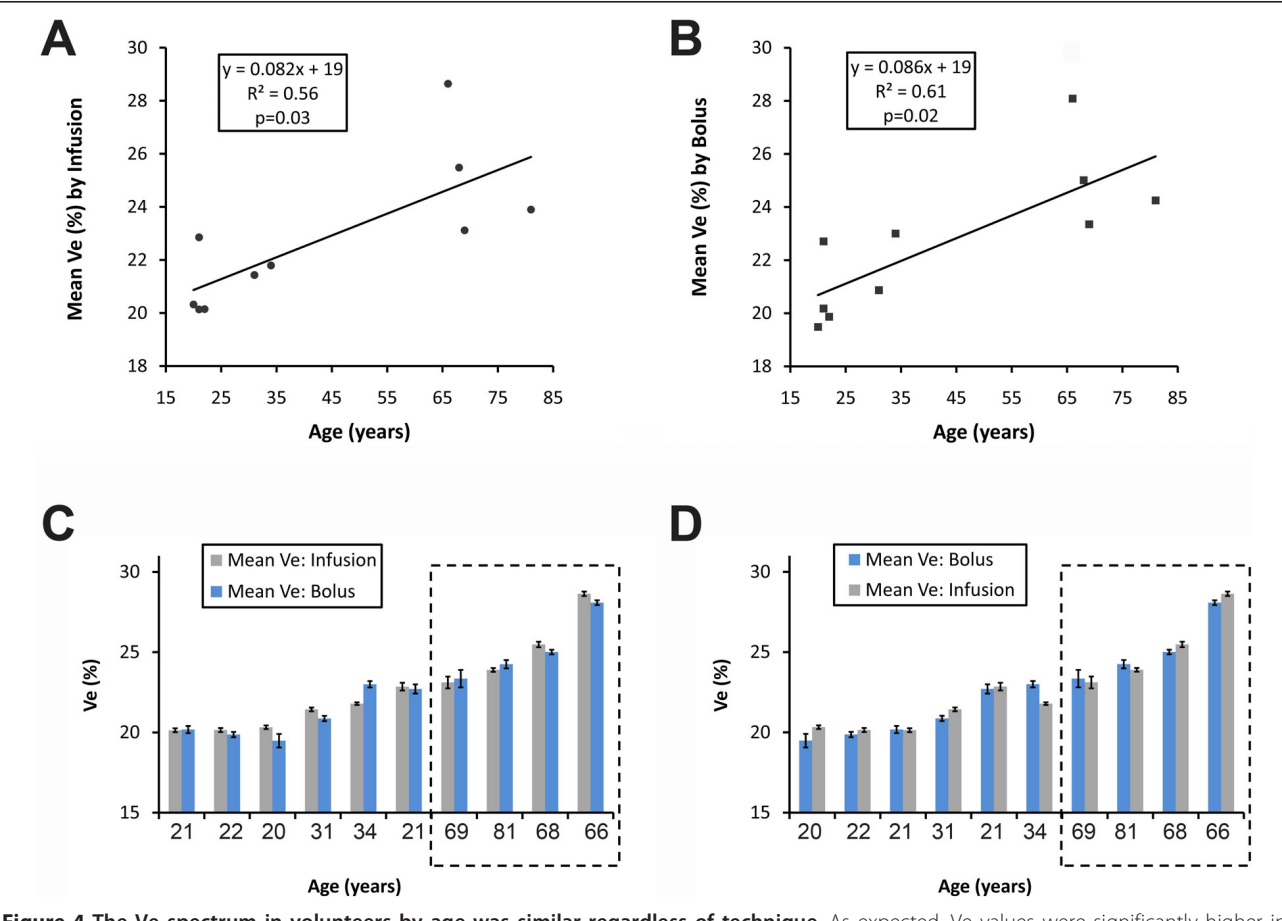
**The Ve spectrum in volunteers by age was similar regardless of technique**. As expected, Ve values were significantly higher in older subjects with significant comorbidity. Ve data from either the infusion technique (Panel A) or the bolus technique (Panel B) yielded similar linear regression results where both techniques detected significantly higher Ve with increasing age. When the same Ve data were then sorted in order of ascending Ve (sorted by Ve from infusion technique in Panel C; sorted by Ve from bolus technique in Panel D), both techniques identically sorted the older subjects with higher mean Ve (dashed boxes). Error bars in the bar graphs indicate the standard error of the mean for repeated Ve measures by each technique in each individual.

Older subjects had higher mean Ve from infusion compared to younger subjects under age 35 (21.1 ± 1.1% vs. 25.3 ± 2.4% by the infusion technique and 20.8 ± 1.8% vs. 25.2 ± 2.1% by the bolus technique, respectively; p ≤ 0.03 for both). Similarly, for each technique, regression analysis of mean Ve values for each subject also yielded a significant relation between Ve and age regardless of whether the infusion technique or the bolus technique was employed (Figure 4). Because heart rate may exert slight effects on T1 measurement by MOLLI, we created a multivariable GEE regression model that adjusted for heart rate. Regardless of technique, Ve remained significantly related to age (p < 0.01 for both), even after adjusting for heart rate which was not significant predictor (p ≥ 0.2). Whether high Ve values were related to age alone or associated comorbidity or both is beyond the scope of this work.

### Comparison of Ve Measurement by Infusion and Bolus Techniques

Baseline *precontrast *T1 measures for the "infusion" scan and the "bolus" scan permit assessment of T1 variability across scans. There was no significant difference between T1 measures of blood (1534 vs. 1502 msec, p = 0.41) and myocardium T1 (946 vs. 948 msec, p = 0.83). Yet, the median of the absolute value of T1 *differences *in individual subjects between the scans was 20 msec for myocardium (2.1% of mean myocardial T1) and 55 msec for blood (3.6% of mean blood T1), indicating variability attributable solely to T1 measurement *between scans*. This precontrast T1 variation across two scans was greater than T1 variation within one scan. For the infusion scan, precontrast blood and myocardium T1 were measured twice. The median of the T1 *differences *in individual subjects for an extra T1 measurement during the infusion scan was 12 msec for myocardium (1.3% of mean myocardial T1) and 20 msec for blood (1.3% of mean blood T1), indicating the variability of T1 measures *within one scan*.

**Figure 5:**
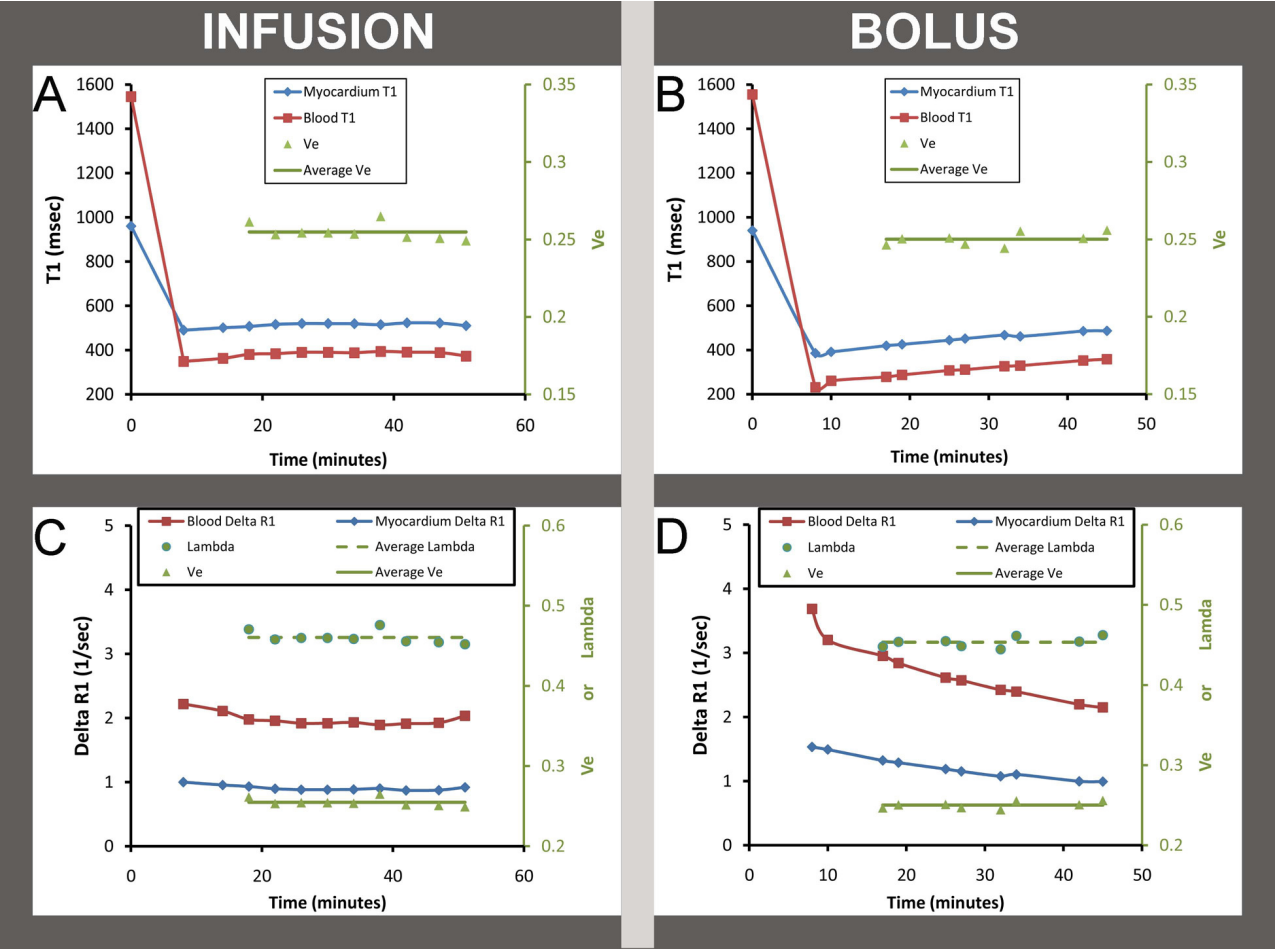
**Ve measurements in a 68 year old female subject with diabetes and hypertension remain constant over time with a constant infusion or with a bolus**. Panel A shows T1 values for blood and myocardium during a constant infusion; the T1 values with associated Ve measures become level and remain relatively constant with <5% variation. Panel B, however shows ever increasing T1 values following a single bolus as gadolinium contrast is cleared. Yet the Ve data (expressed as a proportion, green triangles) yield similar mean Ve values (green line) for each technique. In Panels C and D, the same data (for infusion and bolus, respectively) are expressed as the *change in relaxivity *(delta R1) which linearly relates to gadolinium contrast concentration. T1 and delta R1 do not change rapidly indicating relatively slow clearance. As such, it becomes evident that the ratio of myocardial delta R1 to blood delta R1--the principal determinant of Ve, after adjustment for the hematocrit--remains constant. The ratio is expressed as the partition coefficient lambda (expressed as a proportion, green circles with the green dotted line represents the mean lambda). Indeed, the Ve measures for both the infusion and bolus techniques remain nearly constant at 0.25 (right vertical axis). This finding demonstrates steady state equilibrium between plasma and the myocardial interstitium even as the T1 changes in both tissues during the slow renal clearance of contrast.

Despite the variability in precontrast T1 measurements, Ve data from both methods (i.e., during continuous infusion with steady state defined by stable blood and myocardial T1 and after a simple bolus) yielded similar results. Because the clearance of Gd contrast is slow relative to its equilibration between tissues, both T1 and the ΔR1 changed gradually, and the partition coefficient, λ, remained constant. The stability of infusion- and bolus-based Ve estimation is illustrated in Figure 5. This observation would only occur if steady state between interstitium and plasma was maintained during the clearance of gadolinium contrast. Of note, this 68 year old subject had excellent renal function with a serum creatinine of 0.8 and a preserved glomerular filtrate rate of 71 mL/min/1.73 m^2^.

**Figure 6:**
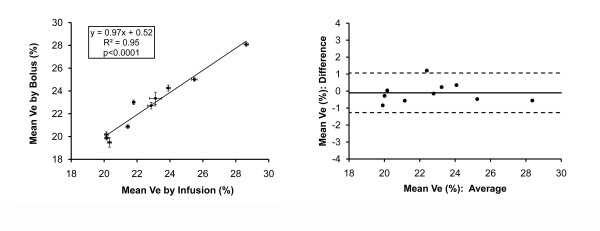
**Correlation and Bland-Altman plots of mean Ve measures for each of the ten subjects by infusion or bolus techniques**. Ve measures by either technique are highly correlated without evidence for significant bias. The correlation plot includes bars representing the mean Ve ± standard error for each subject by bolus or infusion. Bland Altman analysis depicts the mean difference between the bolus or infusion technique (solid line) ± 1.96 SD (dotted lines). The 95% confidence intervals of ± 1.2% for the mean Ve *difference *between bolus and infusion techniques in Bland Altman analysis are comparable to the average 95% confidence intervals for repeated Ve measures by infusion alone (± 1.0%) or by bolus alone (± 1.4%).

With the *infusion technique*, the range of individual Ve measures among subjects was 19.3%-29.2% with a SD≤0.9% for repeated measures (n = 110) in the 10 subjects. Similarly, the range of individual Ve measures among subjects measured by the *bolus technique *was 18.4%-29.1%, with a SD≤1.3% for repeated measures (n = 95). In GEE regression models that adjusted for the clustering of repeated measurements in subjects, serial Ve measures by bolus or infusion did not differ significantly (Ve difference between techniques = 0.1%, p = 0.38). Moreover, when the mean Ve for each subject measured by bolus was plotted against the mean Ve measured by infusion, the regression line had a slope similar to unity, an intercept of nearly zero, and a high R^2 ^value (Figure 6). Importantly, Bland-Altman plots did not reveal significant bias introduced by the bolus technique. Given the lack of a statistically significant difference in Ve measurements related to technique, Ve measurements across CMR scans were therefore reproducible.

The variation between the techniques was similar to the variation within each technique. As shown in the Bland-Altman plots, the 95% confidence intervals of ± 1.2% for difference between mean Ve measures by bolus versus infusion in the were similar to the 95% confidence intervals for repeated measures by either technique. The average 95% confidence interval for Ve measured by infusion was ± 1.0%, and the average 95% confidence interval for Ve measured by bolus was ± 1.4%. Finally, we detected a very small, but statistically significant (p < 0.001) interaction between time and Ve measurement after a bolus. Ve increased slightly by 0.6% over 30 minutes, which was well within the 95% confidence intervals for serial Ve measurements by either infusion (± 1.0%) or bolus (± 1.4%) as shown in Figure 6. There was no time interaction for Ve measures by the constant infusion technique (p = 0.17).

## Discussion

The bolus technique simplifies Ve measurement in humans. With the MOLLI-based T1 computation, Ve can be measured with a simple Gd contrast bolus as accurately as with an infusion, but with slightly less precision, i.e., ± 0.4% absolute difference in the 95% confidence intervals for Ve. Steady state equilibrium is achieved with both methods as evidenced by the stable Ve measures, even as the Gd contrast concentrations changes slowly in myocardium and blood from renal clearance. While we did detect a small, statistically significant time interaction with the bolus technique, the magnitude of the time interaction was well within the 95% confidence intervals for Ve measurement by either technique and therefore of questionable *clinical *significance. Indeed, both methods could easily sort the sample and robustly identify the order of the higher Ve values in older subjects with significant comorbidity, even in the absence of evident late gadolinium enhancement. Since Ve measures obtained from scans performed on different days were not significantly different, Ve measures are therefore reproducible.

The importance of myocardial fibrosis in heart disease has been recognized for decades [1]. A variety of studies indicate that myocardial fibrosis generally represents a final common pathway to a variety of insults [11,35-37]. As such, the human validation study recently published by Flett et al. [14] provides an important tool to those investigating the role of the "interstitial heart disease."[1] Their study established that quantitative Ve measures correlate highly with picrosirius red measures of the collagen volume fraction. Others who employed a bolus strategy for Ve measurement have also shown that Ve values are abnormal in defined patient populations [13,15].

Our work highlights the validity of these previously reported Ve measures based on a bolus strategy [13,15]. We demonstrate that Ve measures are reproducible and reasonably precise from scan to scan with good confidence intervals for Ve estimates, regardless of whether a bolus or infusion technique is employed. We also observe that the range of our Ve data from healthy controls (21.1 ± 1.1% vs. 25.3 ± 2.4% by the infusion technique, and 20.8 ± 1.8% vs. 25.2 ± 2.1% by the bolus technique for young healthy versus older with comorbidity, respectively; p ≤ 0.03 for both) were remarkably similar to the Ve range in controls (mean age was 45 years) reported by Jerosch-Herold et al. [13] (24% ± 3% vs. 31% ± 5% for unaffected family members vs. patients with idiopathic dilated cardiomyopathy, p = 0.002). Similarly, by transforming the "fibrosis index" reported by Broberg et al. [15] to Ve values [where fibrosis index = λ·(1-hematocrit)], we find that healthy controls from this study had Ve values of 21.5% ± 2.1%. Therefore, normal Ve values appear to be approximately 25% or less, but further work is needed to ascertain "normality" for Ve measures. Note, the only difference between Ve as computed both in our manuscript as well as the manuscript by Jerosch-Herold et al. [13] compared to the "myocardial volume of distribution" described by Flett et al. [14] or the "fibrosis index" described by Broberg et al. [15] is that the Ve measure simply multiplies these latter measures by the specific density of myocardium, ρ (1.05), and then subtracts the plasma volume fraction in myocardium which is assumed to be constant at 4.5%.

The bolus strategy to measure myocardial Ve simplifies the Ve data acquisition protocol and facilitates its integration into routine CMR practice. We believe these features will accelerate investigation in the role of the interstitium in health and disease by minimizing the burden on patients and investigators imposed by Ve assessment. Furthermore, the reproducibility of Ve measures between CMR scans is important for studies examining factors that affect changes in Ve over time (e.g., as a result of intervention).

**Figure 7:**
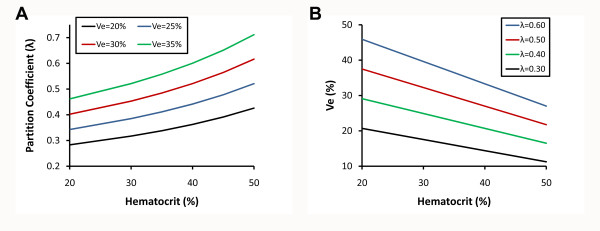
**Hematocrit exerts a significant influence on the relationship between Ve and the partition coefficient, λ**. Ve (expressed as a percentage) is defined here as the following: Ve = [λ · (1-hematocrit) · ρ] - Vp where Ve is the myocardial extravascular extracellular volume fraction, ρ is the specific density of myocardial tissue (1.05), Vp is the myocardial plasma volume fraction (assumed to be a constant 0.045, reflecting capillary density), and λ=[ΔR1_myocardium_]/[ΔR1_bloodpool_] expressed as a decimal). Since anemia is common in various conditions (e.g., heart failure), obtaining the hematocrit is essential for accurate Ve measurement. Panel A shows how λ will vary nonlinearly with the hematocrit for a range of Ve values. Similarly, panel B shows how Ve will vary inversely with the hematocrit for a range of λ values.

In support of our conclusions, Thornhill et al. also found similar results for partition coefficients of infarcted and noninfarcted myocardium measured with either a constant infusion technique or a simple bolus [25]. Our work builds on their results in several ways. First, we used Ve as a myocardial extravascular extracellular space marker which is inherently more accurate than partition coefficients since the latter vary significantly as a function of hematocrit for a given Ve as shown graphically in Figure 7. Similarly, for a measured partition coefficient, the resultant Ve will depend on the hematocrit (Figure 7). Thus, hematocrit variation will confound myocardial fibrosis comparisons across subjects if partition coefficients (or even T1 measures) are employed as surrogate fibrosis measures. Second, we measured the partition coefficient and Ve using actual T1 measurements as opposed to signal intensity surrogates (which do not vary linearly with Gd concentration) generated from pulse sequences (spoiled gradient echo) with lower signal to noise ratios than SSFP [38]. Third, we employed a statistical modeling approach to adjust for clustering of non independent repeated measures in subjects and also identify subtle time interactions. Lastly, we studied a fundamentally different population with a narrower spectrum of myocardial disease characterized by more subtle degrees of myocardial remodeling than frank myocardial infarction. Still, we were nonetheless able to identify higher Ve values from older individuals with comorbidity as compared to the lower Ve values from younger healthy subjects with without known comorbidity.

Ve measurement techniques have been developed specifically to quantify the full spectrum of myocardial fibrosis, including diffuse fibrosis not detectable with conventional LGE images that may be difficult to distinguish from background noise [13-15]. Even though none of the subjects exhibited enhancement in the myocardium used for Ve measurement, both techniques identically stratified older individuals with higher comorbidity based on the higher Ve in older subjects with comorbidity. Although not the primary aim of this study, this specific finding is important because it confirms the remarkably robust ability of CMR to detect age-related change in the interstitium directly, in the absence of frank LGE. Other noninvasive techniques lack this capability. Thus, CMR can demonstrate a spectrum of interstitial changes within the myocardial interstitium itself that otherwise remain unappreciable with conventional imaging techniques. We believe Ve measures will provide an important tool to investigate the relationship between age, myocardial fibrosis, and other age-related changes described previously [23,24,39-41].

Accurate Ve measurement relies heavily on robust T1 measurement. MOLLI techniques with balanced steady state free precession (SSFP) are excellent methods to measure the T1 of myocardium and blood before and after contrast from which Ve is calculated. Use of this pulse sequence yielded excellent agreement to T1 values as measured by spin echo relaxometry at both slower and faster heart rates. Our sampling scheme of the T1 recovery curve intentionally used fewer data points for T1 curve fitting than others [13,20,30]. We attempted to minimize breath hold time for human subjects and also minimize disturbing the recovery of magnetization after inversion pulses by 1) minimizing repetitive inversion pulses generated prior to complete T1 recovery, and 2) minimizing repetitive sampling of the magnetization vector which can lead to systematic errors [42]. In fact, the shorter "hybrid MOLLI" scheme that we employed for Ve measurements in human subjects was based upon better accuracy and precision for T1 measurement in phantoms than the conventional T1 sampling scheme [20]. We note that Piechnik et al. who also have recognized the limitations of "classic MOLLI" have recently published similar accuracy data whereby shorter MOLLI sampling schemes are less heart rate dependent [43]. We also note that their "conditional" T1 sampling approach is conceptually similar to the "hybrid MOLLI" technique whereby sampling is adapted to T1 curve.

We attempted to minimize disturbance of the magnetization vector recovery by employing balanced SSFP. Compared to other T1 measurement techniques that employ spoiled gradient echo readouts, balanced SSFP offers higher signal to noise ratios and minimal disturbance of the magnetization vector that may produce systematic errors in T1 measurement, especially at higher heart rates [19,42]. In addition, SSFP is less susceptible than gradient echo to flow artifact in the blood pool [38] which can also perturb T1 measurements. Indeed, the magnitude of variation in precontrast T1 blood values was not much larger than the variation in precontrast myocardium T1 values. Moreover, MOLLI data can be obtained in a single breath hold, rendering the acquisition fast and patient friendly. In contrast, segmented T1 measurement techniques require 7-8 breath holds per T1 measurement [14]. If an individual is unable to breath hold, MOLLI images can still be obtained without respiratory motion ghosting artifact typical of segmented images (but potentially at the expense of through plane motion). This feature is particularly important in the setting where arrhythmia is present or where patient comorbidity prevents successful breath holding. Unlike segmented T1 measurement techniques [14], MOLLI can be applied to the full spectrum of patients, regardless of comorbidity, thus avoiding potential selection biases. Nonetheless, efforts to optimize T1 measurement strategies upon which Ve is based continue to evolve [43].

Our work has some limitations. First, we could not obtain histologic validation to corroborate our Ve measures because our subjects had no indication for heart surgery or endomyocardial biopsy. Nonetheless, Flett et al. have previously demonstrated excellent correlation of Ve values with the collagen volume fraction in human subjects [14]. They used a different method to measure T1, but we have demonstrated that our T1 measures agree very well with meticulous T1 relaxometry measures obtained from phantoms with representative T1 and T2 values. Second, to compare bolus versus infusion Ve measures, we also did not sample the entire spectrum of myocardial remodeling as evidenced by the absence of late gadolinium enhancement abnormalities in our subjects. We cannot exclude that individuals with very large areas of fibrosis/necrosis with low capillary density might not exhibit steady state pharmacokinetics with a bolus. Yet, Thornhill et al. did not find evidence of different partition coefficients measured with either bolus or infusion techniques within the extremes of grossly infarcted myocardium or apparently normal myocardium [25]. Thus, we believe the simple bolus strategy is valid across the fibrosis spectrum, with one notable exception: systemic amyloidosis. This condition is characterized by marked interstitial expansion from deposition of amyloid proteins and very rapid, presumably extrarenal clearance of Gd contrast from the blood pool [44], and we suspect that a bolus might not yield accurate Ve measures under such conditions. Third, we did not measure renal function in some of the younger subjects although there was no indication of renal impairment. Fourth, our Ve measures excluded the edges of the myocardium due to concerns about partial volume error in the setting of limited spatial resolution [12]. This approach will not detect fibrosis limited to the subendocardium or subepicardium. Myocardial fibrosis in nonischemic cardiomyopathy is a generally diffuse process [11], but ischemic cardiomyopathy demonstrates more fibrosis in the subendocardium [36]. Lastly, we, like other investigators [13-15], did not account for any variation in myocardial plasma volume fraction (Vp) across individuals; this issue requires further study using additional techniques beyond the scope of this manuscript.

## Conclusions

Ve measurement did not differ significantly between techniques employing a simple contrast bolus or a smaller bolus followed by a lengthy infusion to demonstrate steady state. Both methods identified similarly strong relationships between Ve and age. Both methods could easily sort the study sample and robustly identify the order of the higher Ve values in older subjects. Ve measures were also reproducible since we could not identify a statistically significant difference between Ve measures from different scans. Because the bolus strategy simplifies Ve measurement, these data may facilitate integration of Ve measurement into routine CMR practice. This strategy may accelerate investigation of the role of Ve in health and disease by avoiding the burden imposed by an infusion on patients and investigators. The reproducibility of Ve measures across scans is also important for studies examining changes in Ve measures.

## Authors' contributions

All authors read, critically edited the initial manuscript, added intellectual content, and approved the final version. EBS and TCW designed the study, created the phantoms, acquired, and analyzed the phantom data. EBS and BLJ conducted the statistical analyses. EBS conceived of the "hybrid" method, performed all CMR scans, and drafted the initial manuscript. SMT, CGM, WJC, JEL, AJB, analyzed the human data. PK assisted with pulse sequence optimization and wrote the simulation program. DRL modified and performed the final simulation. SGS wrote the fitting algorithm for T1 measures and assisted with data analysis and interpretation. BLJ wrote the code for statistical analysis and assisted with implementation and interpretation. DS assisted with the initial study design, data analysis, and added critical manuscript content.

## Competing interests

Dr. Schelbert has served as an unpaid scientific advisor to Siemens Medical Solutions which provided the MOLLI sequence. The remaining authors declare that they have no competing interests
